# CD8^+^ tumor‐infiltrating lymphocytes contribute to spontaneous “healing” in HER2‐positive ductal carcinoma in situ

**DOI:** 10.1002/cam4.715

**Published:** 2016-04-06

**Authors:** Michi Morita, Rin Yamaguchi, Maki Tanaka, Gary M. Tse, Miki Yamaguchi, Naoki Kanomata, Yoshiki Naito, Jun Akiba, Satoshi Hattori, Shigeki Minami, Susumu Eguchi, Hirohisa Yano

**Affiliations:** ^1^ Department of Pathology and Laboratory MedicineKurume University Medical CenterKurumeFukuokaJapan; ^2^ Department of PathologyKurume University School of MedicineKurumeFukuokaJapan; ^3^ Department of SurgeryNagasaki University Graduate School of Biomedical SciencesNagasakiJapan; ^4^ Department of SurgeryJapan Community Health Care Organization Kurume General HospitalKurumeFukuokaJapan; ^5^ Department of Anatomical and Cellular PathologyThe Chinese University of Hong KongHong KongHong Kong; ^6^ Department of PathologyKawasaki Medical SchoolKurashikiOkayamaJapan; ^7^ Department of Biostatistics CenterKurume University School of MedicineKurumeFukuokaJapan; ^8^ Department of SurgeryNagasaki Harbor Medical Center City HospitalNagasakiJapan

**Keywords:** CD8^+^ tumor‐infiltrating lymphocyte, DCIS, healing, HER2, regression

## Abstract

We evaluated the associations between tumor‐infiltrating lymphocytes (TIL) including CD8‐positive [+] lymphocytes in ductal carcinoma in situ (DCIS) and histopathologic factors, particularly spontaneous “healing” and immunohistochemical (IHC)‐based subtypes, to clarify the effects of host immune response to cancer cells proliferation during early carcinogenesis for the breast cancer. This cohort enrolled 82 DCIS patients. We examined the relationships between clinicopathologic factors including age, DCIS architecture, Van Nuys classification, grade, comedo necrosis, apocrine features, TIL, CD8^+^ lymphocytes, healing, estrogen receptor and HER2 positivity, and IHC‐based subtypes [luminal, luminal‐HER2, HER2‐positive, triple negative (TN)]. The results were analyzed by univariate and multivariate analyses. High numbers of TIL (high‐TIL) and healing were seen in 30.5% and 39.0% of the cohort, respectively. The distributions of luminal, luminal‐HER2, HER2 and TN subtypes were 73.2%, 9.8%, 13.4%, and 3.6%, respectively. High Van Nuys grading, high‐grade, comedo necrosis, apocrine features, high‐TIL, high CD8^+^ lymphocytes and healing were significantly associated with HER2‐positive (luminal‐HER2, HER2), and TN subtypes. High‐TIL was significantly associated with high‐grade, comedo necrosis, apocrine features, healing, high CD8^+^ lymphocytes and HER2 and TN subtypes. Healing was significantly correlated with high CD8^+^ lymphocytes, high‐grade, comedo necrosis, apocrine features, and HER2‐positive and TN subtypes. Logistic regression analysis revealed a strong association between healing and TIL (odds ratio: 11.72, *P *= 0.024). High CD8^+^ lymphocytes was also significantly associated with healing (odds ratio: 9.26, *P *= 0.009). The results of this study suggested that the spontaneous healing phenomenon might be induced by CD8^+^ high‐TIL associated with high‐grade, comedo necrosis, apocrine features and HER2‐positive DCIS.

## Introduction

The “healing” phenomenon was first described by Muir and Aitkenhead in 1934, who reported that “cancer cells are undergoing retrogressive change and disappearing, this being accompanied by fibrous thickening of intra‐duct walls” 1. Rosen also introduced a process referred to as “healing”, stating that “marked periductal fibrosis can, on occasion, be associated with extensive obliteration of ductal carcinoma in situ (DCIS)” 2. Horii et al. found healing in 7% of patients from a group with a variety of different types of breast cancers, especially in high‐grade tumors with comedo necrosis in the intraductal carcinoma foci 3. Chivukula et al. reported healing findings as “regressive changes” in high‐grade DCIS 4, while Bezic also considered that foreign body giant cells in DCIS represented a sign of the healing phenomenon 5. Although the causes of these phenomena remain unclear, healing is generally considered to reflect a host response to the tumor or its products 2, 4, 5. However, the healing phenomenon has received little attention recently, and previous reports have focused on making an accurate diagnosis in cases with healing, as the tumor content may be low and also masked by the associated severe inflammation and fibrosis 2.

Recent genomic analyses have classified invasive breast cancers into several subtypes that can be approximated using surrogate immunohistochemical (IHC) markers in clinical practice: luminal A‐like, luminal B‐like, HER2‐positive, and triple negative (TN) 6. HER2‐positive (Erb‐B2 overexpressing) and basal‐like subtypes originally showed a poorer prognosis than luminal types (hormone receptor‐positive cancers) 7, but the prognosis of HER2‐positive invasive breast cancers has improved dramatically with targeted anti‐HER2 therapies such as trastuzumab 8. While the concept of surrogate subtyping for invasive cancers is well established, surrogate subtyping for DCIS has attracted much less attention, probably as a result of the lower efficacy of targeted therapeutics in DCIS compared with invasive cancers. Currently, clinical trials targeting HER2‐positive DCIS with different anti‐HER2 treatments with or without radiation are underway 9, 10, 11, and may eventually pave the way for less‐traumatic treatments.

Tumor‐infiltrating lymphocytes (TIL) in breast cancer have recently generated much interest as a prognostic factor 12 and predictive factor for neoadjuvant chemotherapy, especially in HER2‐positive subtype and TN cancers 13, 14. TIL are usually associated with HER2‐positive and TN subtypes rather than luminal subtype of invasive breast cancers 15, and the clinical value of TIL (anti‐ and pro‐tumoral) could be subtype dependent 16.

However, the relationship between TIL and DCIS, precursor to invasive breast cancers, has not been described. One of the subsets of TIL is T cells 12, 13, 14 including antitumor T cells (i.e., CD4^+^/CD8^+^), and may represent an effector immune response against cancer cells 12. Although it has been reported that healing occurs in high‐grade cancers with severe inflammation 1, 2, 3, 4, the details of the inflammatory cells have not been described. We therefore investigated the prevalence of healing in DCIS and its relationships with clinicopathologic characteristics, IHC‐based subtypes, and TIL, particularly the cytotoxic (CD8 positive [+]) T cells.

## Materials and Methods

### Subjects

The study cohort included DCIS patients treated at the Japan Community Health Care Organization Kurume General Hospital in 2008 and 2009. All cases with resected materials were retrieved, biopsy and resection slides were reviewed, and the diagnosis of DCIS was confirmed by two pathologists (M.M. and R.Y). All tissue specimens were embedded in paraffin, processed routinely, and 4‐*μ*m sections were stained with hematoxylin and eosin.

All patients who underwent breast‐conserving surgery received radiotherapy (50 Gy total dose). Other patients had mastectomies. Tissue specimens were obtained from these excisions, with subsequent histologic examination with mapping for DCIS and healing.

This retrospective study was approved by the JCHO Kurume General Hospital Ethical Committee (No 143).

### Clinicopathologic factors

Patients’ age (<50 or ≥50 years) was obtained from the medical records. The histologic slides were evaluated for the DCIS architecture (micropapillary, cribriform, solid, papillary, clinging, solid‐papillary, or combination), Van Nuys classification, tumor grade, comedo necrosis, apocrine features, TIL, healing, estrogen receptor (ER) and HER2 positivity, and IHC‐based subtypes.

### Definitions

#### Comedo necrosis and apocrine features

Comedo necrosis was defined as necrotic debris surrounding by viable cancer cells in any architectural patterns, such as solid, cribriform, micropapillary within duct lumina. No requirement was made for a specific amount of high nuclear grade, nor for a minimum amount of comedo‐type necrosis 17. Apocrine features were defined as cancer cells that had enlarged nuclei with prominent nucleoli and either abundant granular or eosinophilic cytoplasm 18.

#### Grade and Van Nuys classification

Nuclear grade was defined as follows 17, 19: (1) low‐grade nuclei 1–1.5 times the diameter of red blood cells with inconspicuous nucleoli and diffuse chromatin; (2) intermediate‐grade nuclei 1–2 times the diameter of red blood cells with coarse chromatin and infrequent nucleoli; (3) high‐grade nuclei >2 times the diameter of red blood cells, with vesicular chromatin, and one or more nucleoli. The overall grading reflected the Van Nuys classification, with group 3 corresponding to high nuclear grade (nuclear grade 3). The remaining non‐high‐grade lesions (nuclear grade 1 or 2) were stratified by the presence (group 2) or absence (group 1) of comedo‐type necrosis 17. Thus, nuclear grades 1 and 2, and groups 1 and 2 in Van Nuys classification are different groups.

#### IHC‐based subtypes

ER expression (clone SP1, Ventana) and HER2 (C‐erbB‐2) (clone 4B5, Ventana) were evaluated using the Ventana I‐VIEW Breast Panel (Ventana). Antigen retrieval was carried out by heating the sections in EDTA (pH 8.5) in accordance with the manufacturer's recommended protocols. IHC and *HER2* Dual In Situ Hybridization (DISH) DNA Probe Cocktail Assay were performed using the fully automated Ventana Benchmark XT (Ventana, Tucson, AZ) staining system. *HER2* DISH is intended to determine *HER2* gene status by calculating the ratio of the *HER2* gene to chromosome 17. The *HER2* and chromosome 17 probes were detected using two‐color chromogenic in situ hybridization in formalin‐fixed, paraffin‐embedded tissue specimens in accordance with the manufacturer's recommended protocols. ER expression was defined as positive (+) if ≥1% of tumor‐cell nuclei were immunoreactive 20. HER2 IHC expression followed CAP/ASCO guidelines 21. Samples with a HER2 protein score of 2+ were retested by *HER2* DISH.

Molecular subtyping using IHC surrogates was classified as follows: (1) ER^+^/HER2^−^ (luminal); (2) ER^+^/HER2^+^ (luminal‐HER2); (3) ER^−^/HER2^+^ (HER2‐positive); (4) ER^−^/HER2^−^ (TN). These are based on the modified St. Gallen recommendation for invasive breast cancers 6 based on IHC staining.

#### TIL

**Figure 1:**
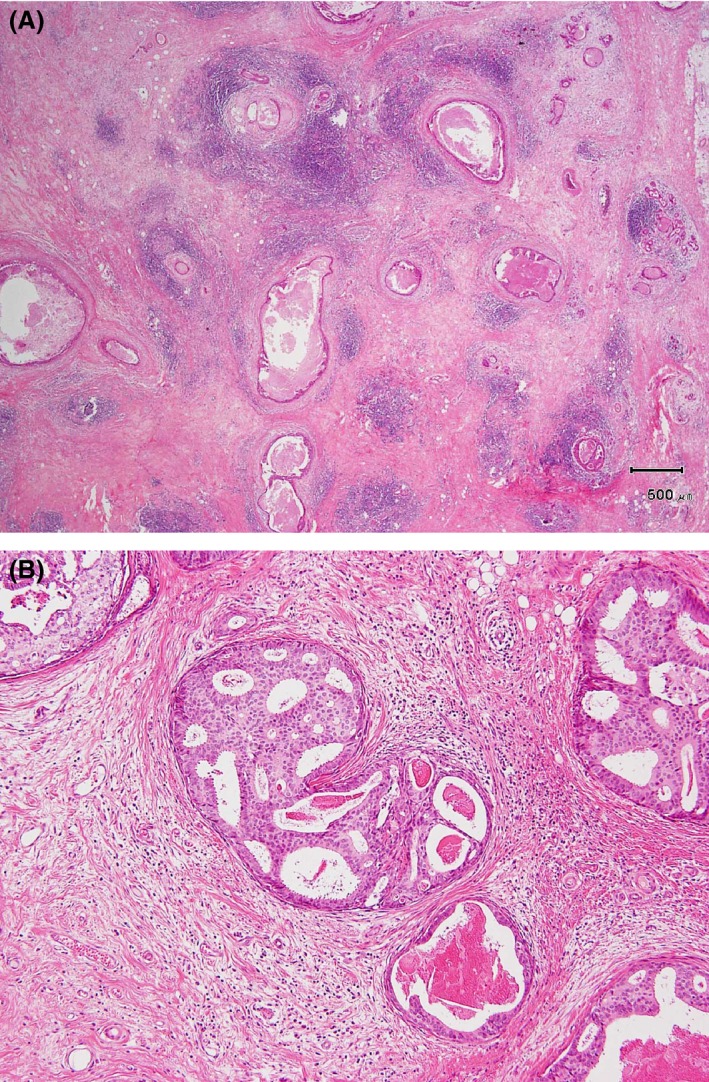
(A) High tumor‐infiltrating lymphocytes (TIL). TIL were observed in the stroma surrounding comedo‐ ductal carcinoma in situ (DCIS) with or without fibrotic changes, as an almost‐complete (>80%) or complete (100%) dense belt of lymphocyte infiltrate surrounding an individual DCIS focus. (B) Low‐TIL was recorded when <50% of the stroma contained lymphocyte infiltrates surrounding DCIS.

TIL were assessed as stromal lymphocytes and stratified as high or low, based on the average and percentage criteria 22. TIL were high (high‐TIL) when >50–100% of the stroma surrounding the DCIS with or without fibrotic changes showed lymphocytic infiltrate (Fig. 1A). Low‐TIL was recorded when <50% of the stroma contained lymphocyte infiltrates (Fig. 1B).

#### CD8^+^ TIL

When the CD8^+^ lymphocytes were identified at low‐magnification, the CD8^+^ lymphocytes were manually counted within a square, 10 mm/10 units by high‐magnification (× 400: 0.0625 mm^2^) at four fields using an eyepiece micrometer (Olympus, Tokyo, Japan).

#### Healing

**Figure 2:**
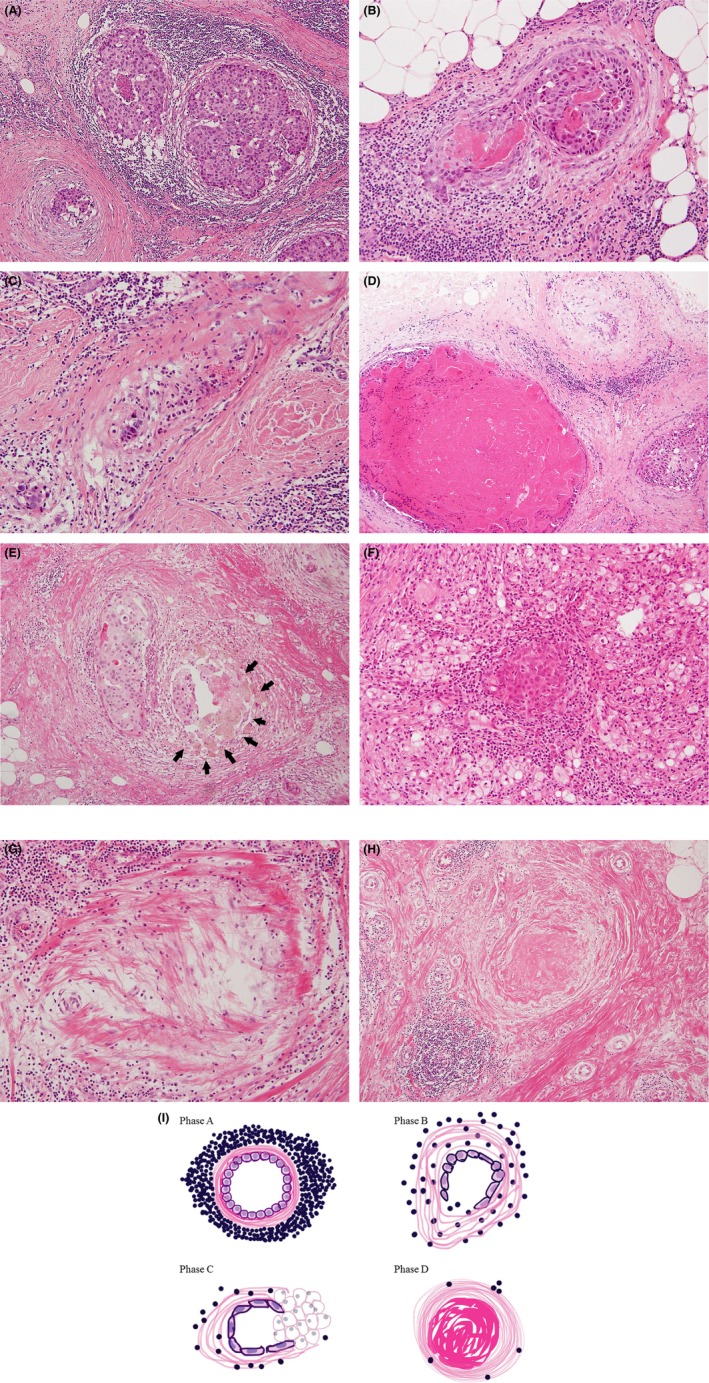
Healing process. Phase A: thin (A) to moderate (B) periductal fibrosis with intratumoral and stromal tumor‐infiltrating lymphocytes (TIL) in high‐grade ductal carcinoma in situ (DCIS) with comedo necrosis and brisk mitoses. Phase B: (C) dense periductal and intraductal fibrosis with residual necrotic DCIS and background TIL; (D) different stages of healing: TIL and fibrous changes surrounding high‐grade DCIS focus with (left bottom) or without (right bottom) a bulky comedo necrosis, and nearly end‐stage healing in an in situ structure (middle top). Phase C: (E) fibrotic changes with histiocytes (arrow) and lymphocytes in situ and surrounding DCIS indicating spontaneous tumor phagocytosis; (F) stromal and intraductal TIL with prominent xanthoma cells induced around a DCIS focus. Phase D: (G) collagen connective fibers increased with lymphocytes in the in situ lesion; (H) a complete scar with elastic fibers was formed with no carcinoma focus in a previous intraductal structure during the final stage of healing; (I) the schema in these phases of healing.

The healing model was modified from previous studies 1, 2, 3, 4, 5: phase A, thin to thick periductal fibrosis with stromal TIL in the DCIS (Fig. 2A and B); phase B, marked circumscribed fibrosis generated around residual cancer cells in DCIS background of TIL, which are less abundant than in phase A (Fig. 2C and D); phase C, histiocytes (xanthoma cells, macrophages, giant cells) containing phagocytosed necrotic cancer cells are seen (Fig. 2E and F); phase D, final stage of scar formation composed of collagen fibers without carcinoma foci in a previous in situ lesion (Fig. 2G and H).

**Figure 3:**
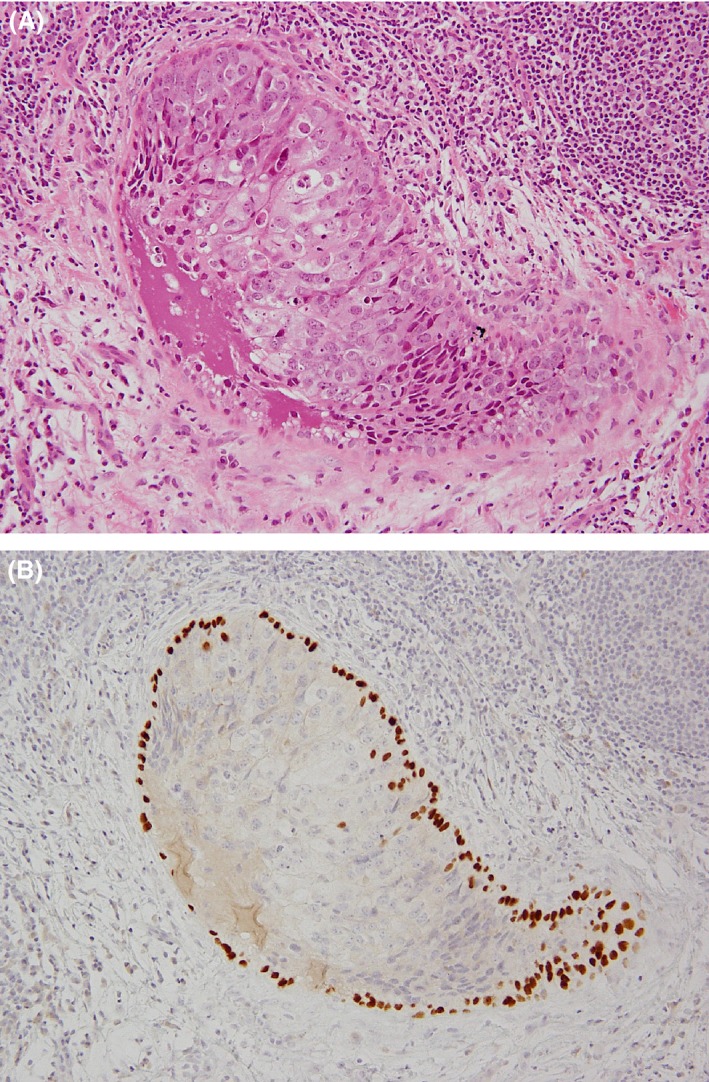
Myoepithelial cells at the site of healing. (A) It is difficult to determine whether it is an in situ lesion or not because of the large number of tumor‐infiltrating lymphocytes with a healing in this H&E stain at a glance. (B) Myoepithelial cells are confirmed by p63 surrounding the duct at the same site, that is, ductal carcinoma in situ.

Except for phase D, a variable number of residual cancer cells may be present in the ductal structures. In this study, the presence of phases A, B, C, or D or any combinations were taken as evidence of healing. Figure 2I shows a schema of these healing phases. The scars by biopsy sites were not considered healing in this study. Some DCISs, which were difficult to distinguish from microinvasive carcinomas, were confirmed with existence of myoepithelial cells using p63 (4A4; Dakocytomation, Carpentaria, CA, USA). (Fig. 3A and B).

### Statistical analysis

The associations between biomarkers and clinicopathologic findings were evaluated using Fisher's exact tests. The association between healing, and TIL and CD8^+^ lymphocytes was examined by logistic regression analysis, adjusting for HER2, ER, grade, and Van Nuys grading, with Firth's correction to avoid unstable estimation. A *P* value of <0.05 was considered significant. Because of the descriptive nature of the study, no multiplicity adjustment was made.

## Results

### Clinicopathologic findings of DCIS

A total of 598 resected breast cancers including 82 (13.9%) cases of DCIS constituted this study cohort. All 82 cases were diagnosed by biopsy, with 46 subsequent partial (breast‐conserving surgery) and 36 total mastectomies. The median number of blocks were 19 (range 4–44) for breast‐conserving surgeries and 3.5 (range 1–16) for mastectomies.

The clinicopathologic findings are shown in Table 1. Combined architecture was seen in 38 cases (46.4%), and the single most common architecture was cribriform (27 cases, 32.9%). According to the Van Nuys classification, 41.5%, 37.8%, and 20.7% of cases were groups 1, 2, and 3, respectively; while 51.2%, 28.1%, and 20.7% were grades 1, 2, and 3, respectively. Comedo necrosis and apocrine features were present in 57.3% and 25.6% of cases, respectively. Low‐TIL occurred in 69.5% and high‐TIL in 30.5% of cases. Healing was present in 39.0% of all cases.

**Table 1:** Clinicopathologic characteristics of 82 cases of ductal carcinoma in situ (DCIS)

	Number of cases (*n *= 82)	(% of patients)
Age (y)		
<50	35	42.7%
≥50	47	57.3%
DCIS structures		
Micropapillary	2	2.4%
Cribriform	27	32.9%
Solid	2	2.4%
Papillary	5	6.1%
Clinging	0	0.0%
Solid‐papillary	8	9.8%
Mixed	38	46.4%
Van Nuys classification		
Group 1	34	41.5%
Group 2	31	37.8%
Group 3	17	20.7%
Nuclear Grade		
Grade 1	42	51.2%
Grade 2	23	28.1%
Grade 3	17	20.7%
Comedo		
Present	47	57.3%
Absent	35	42.7%
Apocrine feature		
Present	21	25.6%
Absent	61	74.4%
Tumor‐infiltrating lymphocytes		
High	25	30.5%
Low	57	69.5%
Healing		
Present	32	39.0%
Absent	50	61.0%
ER		
Positive	68	82.9%
Negative	14	17.1%
HER2		
Positive	19	23.2%
Negative	63	76.8%
Biology‐based tumor types		
HR^+^/HER2^−^(luminal)	60	73.2%
HR^+^/HER2^+^(luminal‐HER2)	8	9.8%
HR^−^/HER2^+^(HER2)	11	13.4%
HR^−^/HER2^−^(Triple negative)	3	3.6%

Among the 32 cases with evidence of healing, the healing phases (A, B, C, and D) were either singular or combined, with phase A, phase A + B, phase A + C, phase A + B + C, phase A + B + D, phase A + B + C + D, and phase B in 10 (31.3%), 10 (31.3%), three (9.4%), five (15.6%), one (3.1%), two (6.2%), and one (3.1%) cases, respectively. Regarding IHC‐based subtyping, the proportions of luminal, luminal‐HER2, HER2‐positive, and TN subtypes were 73.2%, 9.8%, 13.4%, and 3.6%, respectively.

### Relationship between IHC‐based subtypes and histopathological features

We investigated the relationships between histopathologic features and IHC‐based DCIS subtype (Table 2). The median number of CD8^+^ lymphocytes was 87 (range: 1–569). Thus, we divided these into two groups; CD8^+^ high (CD8^+^ was 87 or more) and CD8^+^ low (less than 87). High Van Nuys classification score and high‐grade, comedo necrosis, apocrine features, high‐TIL, high CD8^+^ lymphocytes, and healing were significantly associated with HER2‐positive (luminal‐HER2, HER2 type) and TN breast cancers.

**Table 2:** Relationships between tumor types and clinicopathologic characteristics

	ER^+^		ER^−^		*P*‐value
	HER2^−^	HER2^+^		HER2^−^	
	(*n *= 60)	(*n *= 8)	(*n *= 11)	(*n *= 3)	
Age (y)					N.S.
<50	28	3	2	2	
≥50	32	5	9	1	
DCIS structures					N.S.
Micropapillary	1	0	1	0	
Cribriform	20	2	3	2	
Solid	0	0	2	0	
Papillary	5	0	0	0	
Clinging	0	0	0	0	
Solid‐papillary	7	1	0	0	
Mixed	27	5	5	1	
Van Nuys classification					*P* < 0.001
Group 1	33	0	1	0	
Group 2	24	3	2	2	
Group 3	3	5	8	1	
Nuclear grade					*P* < 0.001
Grade 1	41	0	1	0	
Grade 2	16	3	2	2	
Grade 3	3	5	8	1	
Comedo					*P* < 0.001
Present	26	8	10	3	
Absent	34	0	1	0	
Apocrine feature					*P* < 0.001
Present	7	5	7	2	
Absent	53	3	4	0	
Tumor‐infiltrating lymphocytes					*P* < 0.001
High	8	6	8	3	
Low	52	2	3	0	
CD8 positive lymphocyte					*P* < 0.001
High	22	8	8	3	
Low	38	0	3	0	
Healing					*P* < 0.001
Present	14	7	8	3	
Absent	46	1	3	0	

### Relationships between TIL and histopathologic characteristics

**Figure 4:**
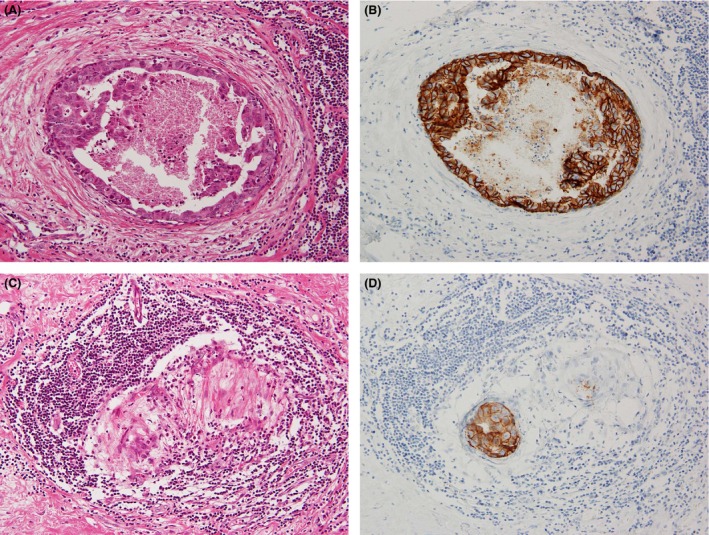
Relationships among tumor‐infiltrating lymphocytes (TIL), healing, and HER2 ductal carcinoma in situ (DCIS). (A) High‐grade comedo‐DCIS with healing and high‐TIL. (B) HER2 was overexpressed in comedo‐DCIS, corresponding to Figure 4A. (C) Healing was associated with high‐ TIL surrounding the residual intraductal carcinoma. (D) HER2 was overexpressed in the residual DCIS, corresponding to Figure 4C.

**Figure 5:**
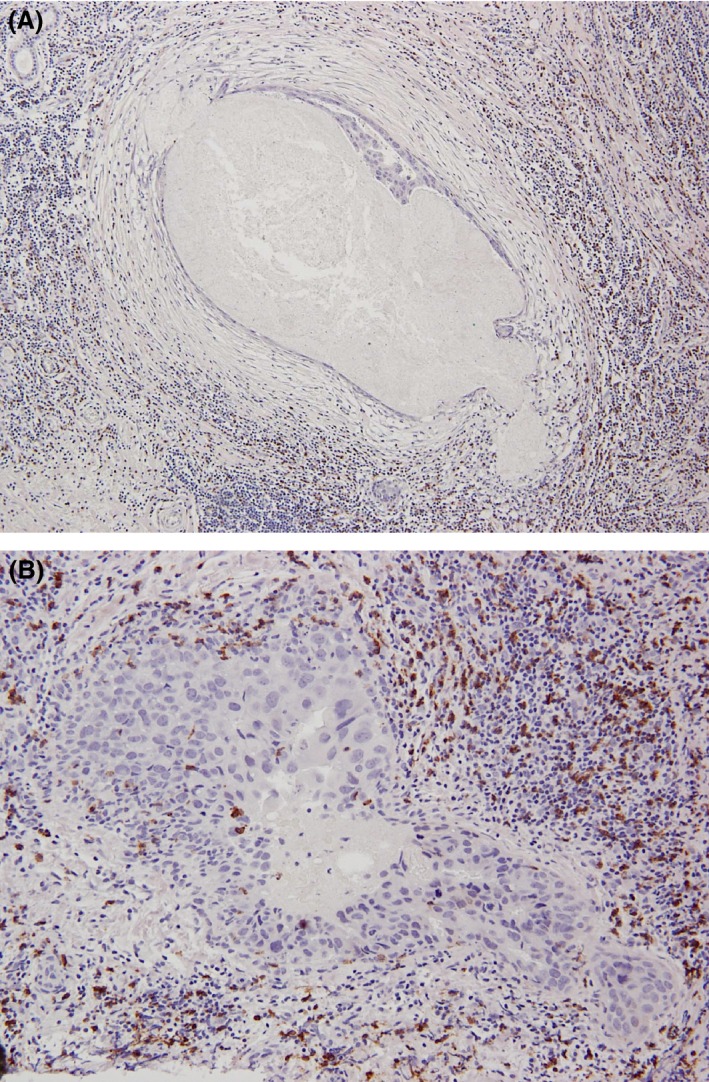
Cytotoxic T cell, CD8^+^ lymphocytes in high‐ tumor‐infiltrating lymphocytes (TIL). (A) CD8^+^ lymphocytes in high‐TIL were seen surrounding ductal carcinoma in situ with healing. (B) CD8^+^ lymphocytes in high‐TIL were seen in not only periductal (stromal) carcinoma foci, but also in intraductal carcinoma foci.

The results of univariate analysis of the relationships between TIL and histopathologic findings in DCIS are shown in Table 3. High‐TIL was significantly correlated with histologic features except for patient age and DCIS structure. High‐TIL was positively associated with high tumor grade, presence of comedo necrosis, apocrine features, healing, high CD8^+^ lymphocytes**,** and HER2 and TN molecular subtypes (Figs. 4 and 5).

**Table 3:** Relationships between tumor‐infiltrating lymphocytes and clinicopathologic characteristics

	High‐TIL(*n *= 25)	Low‐TIL(*n *= 57)	*P*‐value
Age (y)			N.S.
<50	10	25	
≥50	15	32	
DCIS structures			N.S.
Micropapillary	1	1	
Cribriform	10	17	
Solid	1	1	
Papillary	0	5	
Clinging	0	0	
Solid‐papillary	0	8	
Mixed	13	25	
Van Nuys classification			<0.001
Group 1	3	31	
Group 2	7	24	
Group 3	15	2	
Nuclear grade			<0.001
Grade 1	3	39	
Grade 2	7	16	
Grade 3	15	2	
Comedo			<0.001
Present	22	25	
Absent	3	32	
Apocrine feature			<0.001
Present	18	3	
Absent	7	54	
Healing			<0.001
Present	24	8	
Absent	1	49	
CD8 positive lymphocytes			<0.001
High	25	16	
Low	0	41	
ER			<0.001
Positive	14	54	
Negative	11	3	
HER2			<0.001
Positive	14	5	
Negative	11	52	
Biology‐based tumor types			<0.001
ER^+^/HER2^−^(luminal)	8	52	
ER^+^/HER2^+^(luminal−HER2)	6	2	
ER^−^/HER2^+^(HER2)	8	3	
ER^−^/HER2^−^(Triple negative)	3	0	

### Relationships between healing and histopathologic characteristics

There was a significant correlation between high‐TIL and healing. Healing was also correlated with high tumor grade, comedo necrosis, apocrine features, high CD8^+^ lymphocytes**,** HER2 expressing (luminal‐HER2 and HER2‐positive) subtypes and TN breast cancers (Table 4). Logistic regression analysis revealed a strong association between healing and TIL (odds ratio: 11.72, 95% confidence interval: 1.39–99.12, *P* < 0.024) after adjustment for ER, HER2, nuclear grade, and Van Nuys classification (Table 5). High CD8^+^ lymphocytes were also significantly associated with healing (odds ratio: 9.26, 95% confidence interval: 1.77–48.54, *P* = 0.009).

**Table 4:** Relationships between healing and histopathologic characteristics

	Healing^+^(*n *= 32)	Healing ^−^(*n *= 50)	*P*‐value
Van Nuys classification			<0.001
Group 1 (comedo absent)	6	28	
Group 2, 3 (comedo present)	26	22	
Nuclear grade			<0.001
Grade 1, 2	17	48	
Grade 3	15	2	
Apocrine feature			<0.001
Present	17	4	
Absent	15	46	
CD8 positive lymphocytes			<0.001
High	30	11	
Low	2	39	
ER			<0.001
Positive	21	47	
Negative	11	3	
HER2			<0.001
Positive	15	4	
Negative	17	46	
Biology‐based tumor types			<0.001
ER^+^/HER2^−^(luminal)	14	46	
HER2^+^(luminal‐HER2, HER2)	15	4	
ER^−^/HER2^−^(Triple negative)	3	0	

**Table 5:** Logistic regression analysis of factors associated with healing

	Odds ratio	95%Confidence interval		*P*–value
Estrogen receptor (positive [*n*; 68]/negative [*n*; 14])	0.71	0.05	9.66	0.798
HER2(positive [*n*; 19]/negative [*n*; 63])	1.72	0.16	18.10	0.652
Tumor‐infiltrating lymphocytes(high [*n*; 25]/low [*n*; 57])	11.72	1.39	99.12	0.024^a^
Van Nuys classification(Group 2, 3 [*n*; 48]/Group 1 [*n*; 34])	1.92	0.41	9.09	0.410
Nuclear grade (Grade 3 [*n*; 17]/Grade 1, 2 [*n*; 65])	0.68	0.05	9.56	0.777
CD8 positive lymphocytes (high [*n*; 41]/low [*n*; 41])	9.26	1.77	48.54	0.009^a^

a *P *< 0.05.

**Figure 6:**
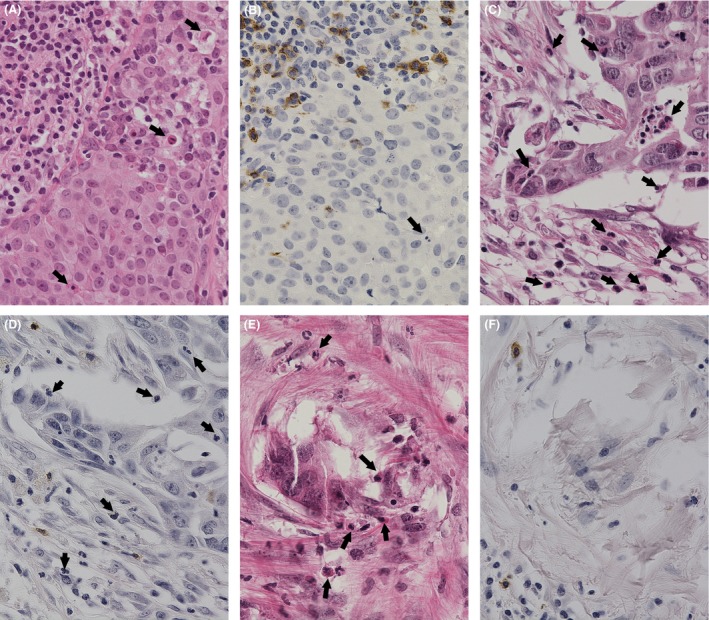
Apoptotic cells and CD8^+^ lymphocytes in each healing phase. Phase A; (A) ductal carcinoma in situ (DCIS) (right lower part) with abundant tumor‐infiltrating lymphocytes (left upper part). Several apoptotic cells which have fragmented nuclei and condensed basophilic cytoplasm are seen in DCIS (arrow). (B) Many CD8^+^ lymphocytes infiltrate into DCIS foci. Apoptotic cells are also seen (arrow). Phases B and C; (C) Striking apoptotic cells (arrow) are seen around DCIS with foci of fibrous tissue; (D) CD8^+^ lymphocytes, apoptotic cells, and viable cancer cells are intermixed in peripheral area of DCIS. (E) In the later stage of healing, carcinoma foci almost disappear and many apoptotic cells are seen. (F) The site is replaced by fibrotic tissue. Not only cancer cells but also CD8^+^ lymphocytes have almost disappeared.

Apoptotic cells with CD8^+^ lymphocytes were also seen in each of the healing phases which are shown in Figure 6A–F.

## Discussion

The results of this study indicated a strong association between spontaneous healing and high‐TIL in patients with DCIS. Although the healing phenomenon in breast cancer has been recognized since 1934 1, it received little attention in routine pathology because its main findings are periductal fibrosis and scars. Healing is associated with severe inflammation 2, which may be the result of the healing process, manifesting as high‐TIL. Bezic 5 suggested that the presence of foreign body giant cells in DCIS was a sign of healing. Although there are possibilities that deeper sections would result in change in the phase of healing, our results suggest that histiocytes, macrophages and xanthoma cells in or around DCIS foci with fibrous change and/or lymphocytes may also denote the healing phenomenon. In the present study, nearly two–thirds of the cases in healing phenomena were seen as combined of phases. The C‐phase of healing (i.e., histiocytes, macrophages) did not exist independently. Futhermore, similar phenomena of spontaneous regression and regressive changes with heavy lymphocytic infiltration and scattered pigment‐containing macrophages have been observed in malignant melanomas 23, 24.

Although DCIS has a better prognosis than invasive breast cancers, healing in this study correlated with poorer histologic features such as high‐grade, comedo necrosis, and predominantly HER2‐positive DCIS. Healing seems a host‐response sign; however, there were not any complete regression cases in our series through the biopsy and the subsequent resected materials. Thus, great care should thus be taken with the residual high‐grade DCISs when they are accompanied with healing and are sometimes masked by high TIL. In this study, healing phenomenon was also seen in low‐TIL in addition to high‐TIL DCIS. In those low‐TIL cases, the TIL existed sporadically around healing ducts, but the quantity did not reach the selection criteria (50%). It might also be possible that TIL disappeared as completed healing represented a late stage event.

TIL and CD8^+^ lymphocytes were significantly related and healing was also significantly associated with TIL and CD8^+^ lymphocytes by the univariate and multivariate analyses. Although intratumoral (intraductal) lymphocytes were not evaluated in this study, intratumoral (intraductal) CD8^+^ TIL was often detected as well as periductal (stromal) CD8^+^ TIL at the site of healing. In addition, existence of apoptotic cells in healing sites exemplified the cytotoxic function of CD8^+^ lymphocytes in inducing apoptosis in its target cells. Thus, periductal CD8^+^ TIL with healing also indicate anti‐tumoral effect. Taken together, we suggest that healing represents a TIL, predominantly CD8^+^ lymphocytes‐mediated host response to carcinoma.

Denkert et al. 13 reported a strong association between TIL and chemotherapy response in invasive breast cancers. TIL have also been considered to be a prognostic factor in TN cancers 25. It has also been reported that CD8^+^ lymphocyte infiltration is an independent predictive factor for pathological complete response to chemotherapy in invasive breast cancer 26 and favorable prognostic indicator in basal‐like breast cancer 27. Few reports have evaluated TIL in DCIS, however, some reports have indicated that TIL may also manifest as stromal or periductal inflammation 4, 28. Our data showed that high‐TIL including high CD8^+^ lymphocytes around DCIS foci was significantly correlated with HER2‐positive and TN DCIS. Interestingly, high‐TIL in invasive cancers was more common in HER2‐positive and TN compared with luminal breast cancers 15. Although the number of TN DCISs in this study is too small to generalize, distribution of TIL among DCIS subtypes were similar to it among invasive carcinoma subtypes. TIL including CD8^+^ lymphocytes could be subtype dependent and may thus play a more significant role in tumor progression from DCIS to invasive cancers in HER2‐positive and TN compared with luminal cancers.

The prevalence of different molecular subtypes differed slightly between DCIS and invasive breast cancers. The incidence of TN DCIS was only 3.6% compared with 10–17% for the TN subtype of invasive breast cancer 29, 30, 31, 32, 33. The same tendency of less TNs compared with other subtypes was seen between 2008 and 2009 (data are not shown). The reported prevalence of TN or basal‐like DCIS was 4–7% 33, 34, 35, 36, with the lowest reported rate of 4.1% from Japan 36. Similarly, our data showed that 51% of DCIS cases were nuclear grade 1 and also showed the relatively greater proportion of luminal DCISs compared to other studies 33, 34, 35. One of the possibilities may be due to our institutional screening center and early detection is made. Another explanation is that the population of DCIS subtypes in Japanese is different than other countries suggested as above. In fact, another Japanese group showed the greater population of luminal (ER+/HER2‐) DCISs [71.9% (282/392)], which was similar to our data. Thus, the proportion of DCIS subtypes in Japan compared with Western populations may be different 35 that TN DCIS cases are less and luminal DCIS cases are more.

The proportion of HER2‐positive DCIS (23.2%) was similar to that of HER2‐positive invasive breast cancers (15–25%) 21, 29. Interestingly, clinical trials of anti‐HER2 therapies have been initiated for HER2 DCIS 9, 10, 11. The anti‐HER2/neu antibody promotes cooperation between innate and adaptive immunity, warranting the use of combination therapies promoting antibody‐initiated anti‐tumor immune responses 37. We found that the correlation between spontaneous healing and HER2‐positive DCIS was associated with CD8^+^ high‐TIL. These results may support that HER2‐positive DCIS has the potential to regress spontaneously with CD8^+^ high‐TIL. Furthermore, high‐TIL surrounding DCIS foci might thus collaborate with anti‐HER2 therapies and immunotherapies to aid the clearance of HER2‐positive DCIS.

Although our sample size including subgrouping was limited, high‐TIL, high‐grade, apocrine features, comedo necrosis, TN and HER2‐positive were significantly associated with healing, with a particularly strong association between CD8^+^ high‐TIL and healing. Spontaneous healing and clearance of DCIS may be associated with high‐TIL as an autoimmune process. This report provides the first evidence for an association between spontaneous healing and TIL, especially CD8^+^ TIL in HER2‐positive DCIS.

## Conflict of Interest

None declared.
